# The Membrane-Associated MARCH E3 Ligase Family: Emerging Roles in Immune Regulation

**DOI:** 10.3389/fimmu.2019.01751

**Published:** 2019-07-24

**Authors:** Heng Lin, Shu Li, Hong-Bing Shu

**Affiliations:** Department of Infectious Diseases, Frontier Science Center for Immunology and Metabolism, Medical Research Institute, Zhongnan Hospital of Wuhan University, Wuhan University, Wuhan, China

**Keywords:** MARCH proteins, E3 ligase, ubiquitination, immune regulation, immune receptors

## Abstract

The membrane-associated RING-CH-type finger (MARCH) proteins of E3 ubiquitin ligases have emerged as critical regulators of immune responses. MARCH proteins target immune receptors, viral proteins as well as components in innate immune response for polyubiquitination and degradations via distinct routes. This review summarizes the current progress about MARCH proteins and their regulation on immune responses.

## Introduction

Ubiquitination is one of the most common post-translational modifications, which plays a key role in regulating stability, localization, protein-protein interaction, and other properties of the substrates. Protein ubiquitination is carried in a multiple step process by the concerted action of E1 ubiquitin-activating enzyme, E2 conjugating enzyme, and E3 ubiquitin ligase. In the final step, the ubiquitin is ligated to a primary amine (i.e., that of lysine, cysteine, serine, threonine or the N-terminus) of substrate proteins by an E3 ligase. In most cases, E3 ubiquitin ligases dictate the specificity of substrates, therefore, are heavily studied.

Based on the structural properties, the E3 ubiquitin ligases are classified into three major types, including the really interesting new gene (RING), the homologous to the E6-AP carboxyl terminus (HECT), and the RING-between-RING (RBR) types of E3 ligases. The RING E3 ligases contain a characteristic RING finger domain, in which eight cysteine and histidine residues coordinate two Zn atoms in the interior of the protein (1).

Membrane-associated RING-CH-type finger (MARCH) proteins are a subfamily of the RING-type E3 ubiquitin ligases (2). MARCH proteins contain a C4HC3-type RING domain that has minor difference with the classic C3HC4-type RING domain in the identities of the fourth and fifth coordinating residues and the length of the peptide segments between the two (3). The MARCH proteins are originally identified as the mammalian structural homologs of the viral immunosuppressive membrane ubiquitin ligases K3 and K5 of Kaposi's sarcoma-associated herpesvirus (KSHV) (4, 5). K3 and K5 of gamma-2 herpesviruses and poxviruses, contained an N-terminal RING-CH domain followed by transmembrane domains, are found to down-regulate the surface expression of major histocompatibility complex I (MHC-I) (6–9). The first identified MARCH protein is c-MIR (now called MARCH8), which was identified by blast searches of the human genome databases (4). c-MIR is a functional homolog of herpesvirus proteins MIR1 and MIR2 and has similar substrate specificity (4). Further bioinformatics studies identified 10 more mammalian MARCH family members, which all possess RING-CH domains with E3 ubiquitin ligase activity.

Recent studies have demonstrated that MARCH proteins are critical regulators of immune responses, which act by catalyzing polyubiquitination of various immune receptors or certain organelle membrane-associated components involved in innate immune responses (3, 10). This review summarizes recent advances in our understanding of properties of MARCH proteins and their emerging roles in regulation of immune responses.

## Properties of the MARCH E3 Ligase Family Members

### Structures of MARCH Proteins

**Figure 1:**
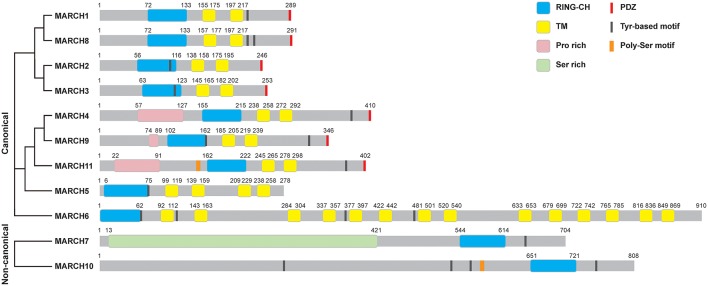
Structures of MARCH proteins. The domain organization for MARCH family members is shown. RING-CH, RING-CH finger domain; TM, transmembrane domain; PDZ, PDZ-binding domain; Pro rich, proline rich domain; Ser rich, serine rich domain; Tyr-based motif, tyrosine-based motif; Poly-Ser motif, poly-serine motif. The phylogenetic tree of MARCH proteins was generated with Clustal Omega alignment using the protein sequences in the Uniprot database.

The most prominent properties of the MARCH family are the RING-CH domain and the transmembrane domains. Except for MARCH7 and MARCH10, the majority of 11 mammalian MARCH proteins share a similar structure, including an N-terminal RING-CH finger and two or more TM domains (Figure 1). MARCH7 and MARCH10 have no recognizable TM spans, with their RING-CH domains located at the C-terminus (3). Therefore, MARCH7 and MARCH10 represent two non-canonical members of the MARCH family. Phylogenetic analysis suggests that the TM-containing MARCH proteins can be classified into several sub-groups, including MARCH1/8, MARCH2/3, MARCH4/9/11, MARCH5, and MARCH6 sub-groups (Figure 1).

Some MARCH proteins harbor other functional domains, such as C-terminal PDZ-binding domains that mediate protein interactions, and tyrosine-based YXXΦ (where X and Φ represent any amino acid and hydrophobic residues, respectively) motifs that are involved in endocytosis (Figure 1). MARCH11 mutant lack of the PDZ-binding domain does not interact with the PDZ-containing protein Veli3, suggesting that its PDZ-binding domain is crucial for its interaction with Veli3, which is important for its transport and subcellular localization (11). MARCH4, 9, and 11 possess an N-terminal proline rich domain that mediates protein interactions. MARCH7 contains an N-terminal disordered serine-rich region, which possesses high sequence similarity to serine/arginine-rich (SR) proteins and contains RNA recognition motifs. MARCH7 is localized on nuclear speckles, a site of the pre-mRNA processing and mRNA export, suggesting a potential role of MARCH7 in these processes (12, 13).

### Subcellular Localizations of MARCH Proteins

MARCH proteins are located in distinct cellular compartments, including the endosomes, lysosomes, endoplasmic reticulum (ER), Golgi apparatus, cytosol and plasma membrane. The localizations of MARCH proteins have direct effects on their functions. MARCH1, 2, 3, 8, and 9 are mostly located at the endosomes, lysosomes and plasma membrane (4, 14–22). Increasing evidences have shown that the localization of the MARCH proteins are important for their roles in regulation of immune receptors. For examples, a study using a chimeric CD86 protein has shown that CD86 is ubiquitinated by MARCH8 at the plasma membrane and followed by being internalized from the plasma membrane (23). MARCH1 is localized on LAMP-1-positive late endosomes/lysosomes, which is consistent with the finding that MARCH1 mediates ubiquitination and lysosome-dependent degradation of MHC class II molecules (24, 25). MARCH3 and MARCH8 regulate interleukin-1 (IL-1) receptor complex-mediated signaling (26). MARCH4 is located on the Golgi apparatus (15). MARCH5 is a mitochondrial outer membrane protein, which regulates mitochondrial morphology and immune response (27, 28). MARCH6 is an ER-resident ubiquitin ligase involved in ER-associated degradation (ERAD) (18). MARCH10 is localized in the cytosol (29). MARCH11 is localized on the multi-vesicular bodies (MVBs) and *trans*-Golgi network (TGN) (11). The PDZ-binding domain of the MARCH proteins plays a role in dictating their subcellular localizations. Mutations of the PDZ-binding domain of MARCH2 and 3 lead to their retention at the ER (16, 17).

### Expression of MARCH Proteins

Most MARCH proteins are ubiquitously expressed in various tissues (Table 1), whereas MARCH1 is strictly expressed in the secondary lymphoid tissues and MARCH11 is mostly expressed in the testis (11, 15, 39). It has been reported that MARCH1-3, 5, 7–9 are highly expressed in immune cells, such as T cells, B cells, monocytes, macrophages and dentritic cells (DCs) (4, 25, 27, 30–32, 39, 44, 45, 49), implying for their potential roles in immune regulation.

**Table 1:** Key features of MARCH proteins.

**Name**	**Localization**	**Expression**	**Function**	**References**
MARCH1 (RNF171)	Lysosome endosome	Secondary lymphoid B cell monocyte immature DC	DC maturation Antigen presentation Insulin resistance	(14, 15, 22, 25, 30–35)
MARCH2 (RNF172)	ER Endosome lysosome Plasma membrane	Ubiquitous	Immune response autophagy Endosomal trafficking	(15–17, 36–38)
MARCH3 (RNF173)	Endosome lysosome Plasma membrane	Ubiquitous	Inflammation	(15, 17, 26)
MARCH4 (RNF174)	Golgi	Lung Brain Placenta	Immune regulation	(15)
MARCH5 (RNF153, MITOL)	Mitochondria	Ubiquitous	Mitochondrial morphology ESC pluripotency Innate immunity	(27, 28, 39–42)
MARCH6 (RNF176, TEB4)	ER	Ubiquitous	ERAD	(18, 43)
MARCH7 (RNF177, AXOT)	Cytoplasm Nucleus Plasma membrane	Ubiquitous	Inflammation T cell development Antigen presentation	(12, 44–48)
MARCH8 (RNF178, c-MIR)	Endosome lysosome Plasma membrane nucleus	Ubiquitous	Inflammation T cell development Antigen presentation	(4, 15, 19, 49–53)
MARCH9 (RNF179)	TGN Lysosome	Lung Lymph node Spleen T, B, DC	Immune regulation	(20–22, 39)
MARCH10 (RNF190)	Cytoplasm	Ubiquitous	Spermatogenesis	(29, 39)
MARCH11 (RNF226)	MVB TGN	Testis	Spermatogenesis	(11)

Several studies have shown that the expression of MARCH proteins is regulated upon cellular stimulation. For examples, expression of MARCH1 is up-regulated following TNFα, IL-1β, and TGFβ stimulation (54). The transcription of *MARCH1* gene is induced by IL-10 and LPS in monocytes or DCs, while its expression is decreased during maturation of DCs, indicating that MARCH1 plays important roles in regulating antigen presentation and DC maturation (22, 25, 31, 33, 34, 55). The transcription of *MARCH9* gene is increased in human DCs upon Toll-like receptor (TLR) 3 or 4 activation (22). The transcription of *MARCH2* gene is markedly induced upon human immunodeficiency virus 1 (HIV-1) infection, and MARCH2-deficiency increases HIV-1 infection in Jurkat and 293T cells (36). These studies suggest that MARCH2 plays unique inhibitory roles in HIV-1 infection. The transcription of *MARCH3* gene is dramatically induced by LPS and TLR8 agonist in monocytes (56). Notably, it has been demonstrated that MARCH1 is highly expressed in human hepatocellular carcinoma (HCC) cells (55), MARCH5 is up-regulated in ovarian cancer tissues (28), and MARCH8 is highly expressed in esophageal tumors and associated with tumor aggression (50). These studies suggest that certain MARCH proteins may involve in tumorigenesis.

### Post-translational Modifications (PTMs) of MARCH Proteins

Several studies have shown that MARCH proteins are tightly and delicately regulated by PTMs. The stability of several MARCH proteins is strictly regulated by ubiquitination. For examples, MARCH1 keeps a low protein level in antigen presentation cells (APCs) of human and mice by its TM-mediated dimerization, leading to autoubiquitination and degradation (57). However, it has been demonstrated that both wild-type and catalytically inactive MARCH1 are ubiquitinated in HeLa cells, suggesting that MARCH1 can be ubiquitinated by as yet unidentified E3 ligases (58). The stability of MARCH5-8, and 10 is tightly regulated by their RING-CH finger-mediated atutoubiquitination (4, 12, 15, 18, 27, 29). USP19 has been shown to remove K48-linked polyubiquitin moieties of MARCH6, which protects it from proteasome-dependent degradation (59). USP7 and USP9X deubiquitinate MARCH7, which promotes its stability (12).

The activity of MARCH ligases is regulated by phosphorylation. For example, MARCH3 is kept inactive by TYRO3-mediated phosphorylation in unstimulated cells. Upon IL-1β stimulation, CDC25A dephosphorylates MARCH3, which in turn activates MARCH3 and causes K48-linked polyubiquitination and degradation of IL-1 receptor type I (IL-1RI), leading to inhibition of IL-1β-triggered signaling (26).

## Immune Regulation by MARCH Proteins

### Regulation of MHCs by MARCH Proteins

MHCs are a set of cell-surface antigen-presenting proteins, which bind to pathogen-derived antigens and subsequently present them on the cell surface for recognition by the T cells (60). MHCs are important for the acquired immune system to recognize foreign molecules in vertebrates.

The expression and turnover of MHC-I on the surface of DCs is essential for their ability to activate CD8^+^ T cells (61). It has been demonstrated that several MARCH proteins can down-regulate MHC-I. Overexpression of MARCH9 causes increased endocytosis of MHC-I, and mediates polyubiquitination of MHC-I HLA-2.1 at its C-terminal lysine residues, leading to its lysosomal degradation (15, 22). Notably, knockdown of MARCH9 impairs the translocation of MHC-I from TGN to endosomes (22), indicating that MARCH9 plays a critical role in coordinating MHC-I access to endosomes and MHC-I-mediated antigen presentation. MARCH4 monoubiquitinates MHC-I, leading to endocytosis of MHC-I from cell surface and degradation (15).

MHC-II is essential for development and activation of CD4^+^ T cells (62). The expression of MHC-II is a prominent feature of professional APCs, and its cell surface expression is strictly regulated to efficiently control antigen presentation through endocytosis and subsequent lysosomal degradation (63). Several MARCH proteins regulate antigen presentation of MHC-II to CD4^+^ T cells and DC maturation (64). MARCH8-transgenic mice display impaired functions in antigen presentation and development of regulatory T cells (Tregs), and are resistant to the onset of experimental autoimmune encephalomyelitis (EAE) (53). MARCH8 mediates polyubiquitination of the β-chain of MHC-II at K225, leading to its lysosome-dependent degradation (53). Interestingly, it has been shown that *Salmonella* effector SteD inhibits antigen presentation and T cell activation by targeting MARCH8, which promotes MHC-II polyubiquitination and surface down-regulation (65). It has been reported that *March8*^−/−^ mice display increased cell-surface MHC-II expression in thymic epithelial cells (TECs) and autoimmune regulator medullary (AIRE)^−^ TECs. MARCH8-mediated polyubiquitination of MHC-II is regulated by CD83, which promotes MHC-II expression by impairing the interaction between MHC-II and MARCH1 (33, 52). Similar to MARCH8, MARCH1 has been identified as another physiological E3 ligase for MHC-II in mouse knockout studies (25, 30, 31, 66–68). In immature DCs, MHC-II undergoes sustaining polyubiquitination by MARCH1, leading to its down-regulation of cell-surface expression (25). During DC maturation, polyubiquitination of MHC-II is reduced and subsequently MHC-II accumulates at the cell surface (25). Further studies demonstrate that MHC-II is less polyubiquitinated and more stably expressed on the cell surface in the mature DCs and B cells in *March1*^−/−^ compared with wild-type mice, resulting in enhanced antigen-presenting ability (68–70). Since MARCH1 plays crucial roles in regulating expression of cell-surface MHC-II, itself is tightly regulated at different DC maturation stages. These findings suggest that MARCH-mediated regulation of MHC molecules is important for immune regulation.

### Regulation of the IL-1 Receptor Complex by MARCH3 and 8

The proinflammatory cytokine IL-1 is a central regulator in the initiation of inflammatory and immune responses. It also plays critical roles in the pathogenesis of different diseases, such as cancer, rheumatoid arthritis, neurodegenerative diseases, and atherosclerosis (71, 72). IL-1 consists of two separate ligands, IL-1α and IL-1β (73). Precursor IL-1α (pro-IL-1α) is biologically active, and is cleaved by calpain to generate mature IL-1α. Both forms of IL-1α are present mostly in the cell, unless released after cell death. In addition to binding to the cell-surface receptors, precursor IL-1α can translocate to the nucleus and affect transcription (74). By contrast, pro-IL-1β is biologically inactive and is cleaved by caspase-1 to produce an active protein (75). Although IL-1α and IL-1β share similar biological effects, IL-1β is more abundantly expressed during the early phase of inflammation response and is a major effector of inflammation (76). IL-1β signals through engagement of a membrane-bound receptor complex consisting of two subunits: IL-1RI and IL-1 receptor accessory protein (IL-1RAcP). Binding of IL-1β to IL-1RI initiates a ligand-induced conformational change of IL-1R1 that facilitates its recruitment of IL-1RAcP via Toll/interleukin-1 receptor (TIR)-TIR domain interaction, leading to the formation of an activated receptor complex. The receptor complex recruits the adaptor protein MyD88. MyD88 further recruits IRAK1, IRAK4, and TRAF6 to the receptor complex, where TRAF6 catalyzes K63-linked autoubiquitination to further recruit the TAK1-TAB2-TAB3 complex, leading to activation of the transcription factors NF-κB and AP-1, induction of downstream effector genes and inflammatory responses (77–85).

**Figure 2:**
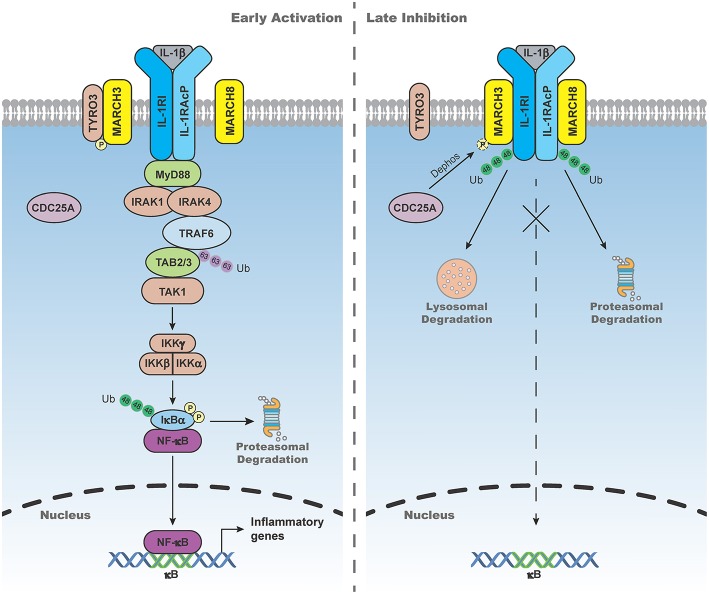
MARCH3- and 8-mediated negative regulation of IL-1β-triggered signaling. In the early phase of IL-1β stimulation, MARCH3 is kept in inactive state by the tyrosine-protein kinase receptor TYRO3-mediated phosphorylation. In the late phase of IL-1β stimulation, MARCH3 is dephosphorylated by cell division cycle 25A (CDC25A), which in turn promotes its E3 ligase activity, leading to K48-linked polyubiquitination of IL-1 receptor type I (IL-1RI) at K409 and its lysosomal degradation. MARCH8 mediates K48-linked polyubiquitination of IL-1 receptor accessory protein (IL-1RAcP) at K512 and its proteasomal degradation upon IL-1β stimulation, leading to attenuation of IL-1β-triggered inflammatory response.

IL-1β-triggered signaling is tightly regulated to avoid excessive inflammatory response. Recent studies have shown that MARCH3 and 8 play important roles in terminating IL-1β-triggered inflammatory response (Figure 2). MARCH3-deficiency potentiates IL-1β-induced transcription of inflammatory genes in BMDMs, monocytes and primary mouse lung fibroblasts (MLFs). MARCH3-deficiency also increases the levels of serum inflammatory cytokines, as well as susceptibility to inflammatory death triggered by IL-1β injection or *Listeria monocytogenes* infection. Mechanistic studies indicate that MARCH3 is kept in inactive state by TYRO3-mediated phosphorylation. Upon IL-1β stimulation, MARCH3 is dephosphorylated by CDC25A, which in turn promotes its E3 ligase activity, leading to K48-linked polyubiquitination of IL-1RI at K409 and its lysosomal degradation (26). Another study has demonstrated that MARCH8 mediates K48-linked polyubiquitination of IL-1RAcP at K512 but not IL-1RI after IL-1β stimulation, leading to attenuation of IL-1β-triggered inflammatory response (51). These observations suggest that distinct members of the MARCH family mediate degradation of different components of the IL-1 receptor complex upon ligand stimulation, which ensures precise control of inflammatory response to avoid self-damage.

### Regulation of Other Immune Receptors by MARCH Proteins

MARCH proteins can also regulate stability of other cell-surface immune relevant receptors, such as cluster of differentiation 86 (CD86, also known as B7-2), TNF-related apoptosis inducing ligand receptor 1 (TRAIL-R1; also known as DR4), intercellular adhesion molecule 1 (ICAM-1, also known as CD54), Mult1, Fcγ Receptor IIb (FcγRIIb), and cystic fibrosis transmembrane conductance regulator (CFTR).

#### Regulation of CD86 by MARCH1 and 8

CD86 is an essential immune regulator expressed on APCs and provides costimulatory signals for T cell activation and survival (86, 87). It has been shown that MARCH8 mediates polyubiquitination of CD86 at its C-terminus, leading to its rapid endocytosis and lysosome-dependent degradation (4). It has also been shown that MARCH1 mediates polyubiquitination of CD86 at K267 and its degradation (34). Consequently, CD86 is more stably expressed on the cell surface of *March1*^−/−^ DCs (34), indicating that MARCH1-mediated polyubiquitination of CD86 is a crucial mechanism in regulating antigen presentation by DCs.

#### Regulation of TRAIL-R1 by MARCH8

Binding of TRAIL to TRAIL-R1 induces apoptosis in cancer cells, which contributes to immunosurveillance (88, 89). MARCH8 targets TRAIL-R1 at K273 for polyubiquitination, which causes its lysosome-dependent degradation in breast cancer cells, leading to the inhibition of TRAIL-R1-mediated apoptosis signaling (90).

#### Regulation of ICAM-1 by MARCH9

ICAM-1 is an immune receptor that contains binding sites for a large amounts of immune-associated ligands. ICAM-1 plays important roles in facilitating transendothelial migration of activated lymphocytes across vascular endothelia in processes such as inflammatory responses (91, 92). It has been demonstrated that overexpression of MARCH9 mediates polyubiquitination of ICAM-1 for lysosome-dependent degradation (20). MARCH9 attenuates the oncogenic effects of ICAM-1, leading to the inhibition of migration and invasion in lung adenocarcinoma (LAC) cells (93). Interestingly, MARCH9 down-regulation in LAC correlates with poor clinical outcomes (93).

#### Regulation of Mult1 by MARCH4 and 9

Stimulation of immune cells through NKG2D receptor has been shown to involve in immune responses to infection and malignancy (94). Expression of the self-encoded ligands for NKG2D is tightly regulated to prevent autoimmune disorders. It has been reported that MARCH4 and 9 down-regulate cell-surface expression of Mult1 which is a ligand for NKG2D receptor, and prevents cells from being targeted for lysis by NK cells (95).

#### Regulation of FcγRIIb by MARCH3

FcγRIIb is an FcγR inhibitor that inhibits FcγR-mediated response to antibody-coated tumor cells. Regulation of FcγRIIb expression is crucial in tumor immunotherapy and autoimmune diseases (96, 97). It has been shown that MARCH3 is required for LPS-induced polyubiquitination and down-regulation of FcγRIIb (56). Notably, the ubiquitination of FcγRIIb precedes LPS-induced up-regulation of MARCH3 (56), suggesting that LPS had an earlier effect on MARCH3 activity.

#### Regulation of CFTR by MARCH2

CFTR is a plasma membrane cAMP-regulated chloride channel (98). Defective CFTR triggers aggresome formation and lung inflammation in cystic fibrosis (CF) by ROS-TG2-BECN1-mediated inhibition of autophagy (99, 100). CFTR also plays crucial roles in the progression and metastasis of cancer (101, 102). It has been reported that MARCH2 mediates polyubiquitination and degradation of CFTR, leading to attenuation of CFTR-mediated autophagy in tumor cells. MARCH2 interacts with CFTR via its PDZ-binding domain (37). These studies suggest that MARCH2 is a negative regulator of CFTR-mediated autophagy.

### Regulation of Innate Immune Response by MARCH Proteins

It has been well-established that viral RNAs act as classic pathogen-associated molecular patterns (PAMPs), which are sensed by endosomal TLRs and cytosolic RIG-I-like receptors (RLRs) (103–106). Recognition of viral RNAs by these receptors links them to downstream adapter proteins, including TRIF, MyD88, and VISA (also called MAVS, IPS-1, and Cardif), leading to activation of the kinases TBK1 and IKKβ. These kinases phosphorylate and activate the transcription factors IRF3 and NF-κB, respectively, which cooperatively induce transcription of a set of antiviral genes including type I interferons (IFNs) (83, 107–119).

Several studies have demonstrated that MARCH proteins are involved in regulation of innate antiviral responses. MARCH5 catalyzes K63-linked polyubiquitination of TANK, which potentiates TLR7-mediated NF-κB activation (40). The protein level of VISA is tightly regulated to ensure its proper activation and timely termination of innate antiviral response. At the late phase of viral infection, VISA is phosphorylated at T54 by protein kinase A (PKA), which primes it for K48-linked polyubiquitination and degradation by MARCH5, leading to attenuation of innate immune response (120). The inactive rhomboid protease iRhom2 mediates proteasome-dependent degradation of MARCH5, resulting in the inhibition of virus-triggered degradation of VISA, which ensures proper level of VISA for innate antiviral response (121). These studies suggest that MARCH5 plays critical roles in temporal regulation of innate antiviral response.

MARCH proteins can regulate cellular antiviral response by targeting viral proteins. MARCH2 is induced upon HIV infection, which inhibits HIV-1 replication by mediating degradation of viral envelope proteins (36). It has also been shown that MARCH8 targets cell-surface envelope glycoproteins of HIV-1 for degradation, resulting in the inhibition of HIV-1 infection (49). MARCH8 catalyzes K63-linked polyubiquitination of hepatitis C virus (HCV) nonstructural 2 protein (NS2) and promotes viral assembly and envelopment (122). It has also been shown that MARCH8 is required for infection with Flaviviridae family members, such as HCV, dengue, and Zika viruses (122). These studies suggest that certain MARCH proteins can be potential host targets for antiviral strategies.

It has been shown that MARCH1-deficiency inhibits TLR3/4-mediated transcription of *Tnfa* gene in splenocytes (123). However, another study has shown that MARCH1-deficiency causes increased LPS-triggered production of proinflammatory cytokines, higher NK cell activation, as well as more susceptible to LPS-induced inflammatory death (124). Whether MARCH1 regulates TLR4-mediated inflammatory responses in a cell specific manner needs to be further investigated.

### Functions of the Non-canonical MARCH Proteins

MARCH7 and MARCH10 are the two non-canonical MARCH family members. While it is unknown for the functions of MARCH10, studies have demonstrated that MARCH7 regulates leukemia inhibitory factor (LIF) secretion and inflammasome activation. In response to ConA, CD4^+^ T lymphocytes release LIF, which plays important roles in immune tolerance (125, 126). It has been shown that MARCH7-deficiency potentiates T cell proliferation and T cell-derived LIF secretion upon mitogen stimulation (46), suggesting that MARCH7 plays critical roles in immune tolerance. However, the mechanisms of MARCH7-mediated regulation of LIF secretion remains unclear.

NLRP3 inflammasome is composed of NLRP3, ASC and caspase-1, which is assembled in response to damage-associated molecular patterns (DAMPs) and PAMPs (127). It has been shown that MARCH7 mediates polyubiquitination of NLRP3 for degradation, leading to the inhibition of NLRP3-dependent inflammation triggered by dopamine D1 receptor (DRD1)-cAMP axis (47).

## Concluding Remarks

The MARCH family of E3 ubiquitin ligases is unique in that they are mostly localized at plasma and/or organelle membranes. Most of the MARCH proteins are abundantly expressed in immune cells. This position them well in regulating immune receptors. In recent years, various studies have demonstrated that MARCH proteins target certain immune receptors for K48-linked polyubiquitination and degradation via distinct routes. In certain cases, MARCH proteins can also target the substrates for K63-linked polyubiquitination. In addition to immune receptors, certain MARCH proteins are also involved in regulation of viral proteins as well as components in innate immune responses. Therefore, the functions of MARCH proteins are not limited to immune receptors. Although considerable progress has been made for the functions and mechanisms of MARCH proteins, many outstanding questions remain. For examples, how are substrate specificities of MARCH proteins determined? Why are different subunits of a receptor complex targeted by distinct members of the MARCH family? One example is that the IL-1R complex components IL-1RI and IL-1RAcP are targeted by MARCH3 and 8 respectively. Whether do MARCH proteins play redundant functions in regulation of certain receptors? For examples, whether do MARCH1, 8 and 9 play redundant roles in down-regulation of MHC-II? Whether are other immune receptors regulated by MARCH proteins? How is the activity of MARCH proteins regulated by upstream signaling events? In addition to MARCH3, are other MARCH proteins regulated by phosphorylation or other post-translational modifications? Finally, we still know little about the pathological relevance of MARCH proteins in human. Further investigations into these outstanding questions would contribute to our understanding of the roles and mechanisms of MARCH proteins in physiological and pathological processes.

## Author Contributions

H-BS and SL conceived and designed this review. HL performed manuscript preparation, literature search, and editing. H-BS, SL, and HL wrote the manuscript.

### Conflict of Interest Statement

The authors declare that the research was conducted in the absence of any commercial or financial relationships that could be construed as a potential conflict of interest.
